# Posttranscriptional Regulation by Copper with a New Upstream Open Reading Frame

**DOI:** 10.1128/mbio.00912-22

**Published:** 2022-07-13

**Authors:** Gauthier Roy, Rudy Antoine, Annie Schwartz, Stéphanie Slupek, Alex Rivera-Millot, Marc Boudvillain, Françoise Jacob-Dubuisson

**Affiliations:** a University of Lille, Inserm, CNRS, CHU Lille, Institut Pasteur de Lille, U1019-UMR9017-CIIL-Center for Infection and Immunity of Lille, Lille, France; b Centre de Biophysique moléculaire, CNRS UPR4301, Orléans, France; affiliated with Université d’Orléans, France; Washington University School of Medicine; Washington University School of Medicine

**Keywords:** copper homeostasis, posttranscriptional regulation, upstream ORF, DUF2946 family, *Bordetella pertussis*

## Abstract

Copper is essential to most living beings but also highly toxic and as such is an important player at the host-pathogen interface. Bacteria have thus developed homeostatic mechanisms to tightly control its intracellular concentration. Known Cu export and import systems are under transcriptional control, whereas posttranscriptional regulatory mechanisms are yet to be characterized. We identified a three-gene operon, *bp2923-bfrG-bp2921*, downregulated by copper and notably encoding a TonB-dependent transporter in Bordetella pertussis. We show here that the protein encoded by the first gene, which is a member of the DUF2946 protein family, represents a new type of upstream Open Reading Frame (uORF) involved in posttranscriptional regulation of the downstream genes. In the absence of copper, the entire operon is transcribed and translated. Perception of copper by the nascent *bp2923*-coded protein via its conserved CXXC motif triggers Rho-dependent transcription termination between the first and second genes by relieving translation arrest on a conserved C-terminal RAPP motif. Homologs of *bp2923* are widespread in bacterial genomes, where they head operons predicted to participate in copper homeostasis. This work has thus unveiled a new mode of genetic regulation by a transition metal and identified a regulatory function for a member of an uncharacterized family of bacterial proteins that we have named CruR, for copper-responsive upstream regulator.

## INTRODUCTION

Bacteria have evolved complex mechanisms to respond to changes of their environment, and notably to strictly regulate the availability of necessary but harmful transition metals. Copper is such a metal both essential and harmful to living beings (1). Its properties as a redox cycling metal have been put to use in electron transfer chains as a cofactor of heme-copper oxidases for aerobic respiration, photosynthesis, and denitrification (2). It is also involved in various hydrolytic and redox reactions catalyzed by metabolic enzymes, and in the protection against reactive oxygen species. Its high affinity for organic molecules makes copper very toxic, notably because it destroys iron-sulfur clusters during or after biogenesis and indirectly induces oxidative stress (3). For its capacity to kill microorganisms, copper is notably used in health care settings and agriculture and has become a common pollutant (4–8). Eukaryotic phagocytes in natural milieus (e.g., amoeba) and at the host-pathogen interface notably employ copper to kill microorganisms (9, 10). Life with copper has therefore led bacteria to develop homeostatic mechanisms that strictly control its intracellular concentration (2, 11, 12). Defense systems against copper include export of Cu^1+^ from the cytoplasm or its passivation by sequestration, the detoxification of Cu^1+^ into Cu^2+^ in the periplasm, and its extrusion to the extracellular medium.

Bacteria also need to acquire copper from their environment (13), and the few described copper uptake systems are dedicated to the assembly of specific cuproproteins (14, 15). The expression of homeostasis genes depends on the intracellular copper concentration. Copper controls homeostasis genes through transcriptional regulation with cytoplasmic regulators or two-component systems (11, 16, 17). Although bacteria also make use of posttranscriptional regulatory mechanisms notably based on riboswitches and small RNAs to ensure homeostasis of other transition metals (18–21), such posttranscriptional regulation mechanisms are yet to be characterized for copper.

Bordetella pertussis is a strictly aerobic, Gram-negative bacterium responsible for whooping cough (22). Compared with other betaproteobacteria, B. pertussis has lost most copper resistance mechanisms (23, 24), probably because its specialized lifestyle as a host-restricted pathogen reduces its exposure to copper except when it is phagocytosed, a fate that it strives to avoid (22, 25, 26). Transcriptomic analyses have identified a three-gene operon predicted to participate in copper import in B. pertussis, *bp2923-22-21*, indicating that the bacterium needs to acquire copper in specific circumstances (24). This three-gene operon is downregulated by excess copper in the medium (24). In this study, we characterized its regulation, which is original for transition metals, and revealed a posttranscriptional mechanism involving an upstream ORF widespread among Proteobacteria.

## RESULTS

### Characterization of a Cu-regulated operon harboring a TonB-dependent transporter gene in B. pertussis.

**FIG 1:**
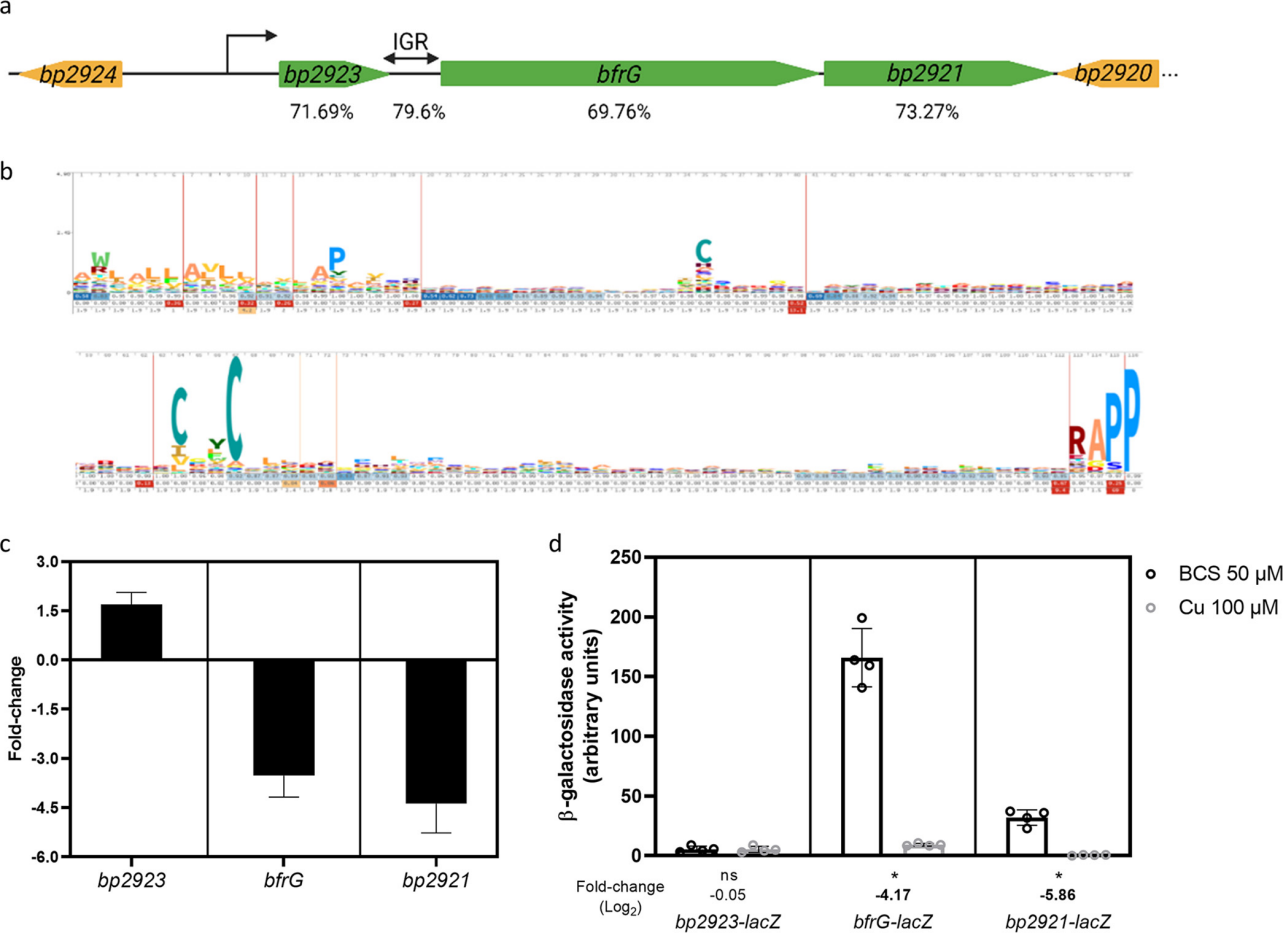
Role of *bp2923* in the regulation of *bfrG* and *bp2921* by copper. a, Schematic representation of the locus in B. pertussis. The transcription start site was identified by 5’RACE analyses. IGR represents the intergenic region between *bp2923* and *bfrG*, and the percentages given underneath the various regions of the operon indicate their (G+C) contents. b, HMM logo of the DUF2946 protein family. Two distinct motifs are well conserved, a putative copper-binding motif CXXC and a C-terminal RAPP motif. Another Cys residue is also semiconserved. c, qRT-PCR analyses showing the ratios of transcription (in log_2_) of each gene in bacteria grown for 16 h in medium supplemented with 100 μM CuSO_4_ relative to bacteria grown in Cu-restricted medium (Cu chelator BCS added to 50 μM). The results were normalized against a housekeeping gene. d, Translational *lacZ* fusions for the three genes. β-galactosidase activities were measured for bacteria grown under the same conditions as in (c), and the individual data points are shown. The bars represent the means of three (panel c) or four (panel d) biological replicates, and the error bars show the standard deviations (SD). Statistics were performed using a nonparametric Mann-Whitney test (*, *P* < 0.05; ns, not significant). In panel d, the expression ratios (in log_2_) of the reporter in bacteria grown for 16 h in medium supplemented with 100 μM CuSO_4_ relative to bacteria grown in medium supplemented with 50 μM BCS are also indicated. Boldfaced letters indicate differences of 4-fold or more between the two conditions.

RNA-seq experiments have identified a three-gene locus, *bp2923-bp2922-bp2921*, of which the last two genes are strongly downregulated by copper (24). The first open reading frame (ORF) is separated from the following gene by a long intergenic region (IGR) of 162 bp. The average (G+C) content of this locus, 71.6%, is higher than that of the B. pertussis genome (Fig. 1a). The three genes form an operon, as shown by RT-PCR on the IGR (Fig. S1).

*bp2923* encodes a putative 145-residue-long protein of unknown function of the DUF2946 Pfam protein family, predicted to be exported. This family is characterized by two conserved sequence motifs, CXXC (where X represent nonconserved residues) and RAPP (Fig. 1b). *bp2922* is predicted to encode a TonB-dependent transporter (TBDT) previously named BfrG (27). TBDTs form a large family of outer membrane proteins mediating import to the periplasm of various types of small molecules and notably iron, in the form of Fe-siderophore complexes or scavenged from host proteins (28). The third gene, *bp2921*, encodes a protein predicted with four transmembrane segments and two periplasmic domains, and that belongs to the PepSY_TM family, a member of which has been described as a siderophore reductase (29). Proteomics analyses of B. pertussis extracts identified peptides of the last two proteins but not of the *bp2923* gene product (24).

10.1128/mbio.00912-22.5 FIG S1 Operonic structure of *bp2923* and *bfrG*. a, Localization of the primers used for the PCR reactions (red arrows). b, The PCR were conducted with the primers shown above to amplify a 458-bp region straddling IGR. Reverse transcription followed with PCR was conducted on DNAse-treated RNA extracted from a midlog-phase culture of BPSM grown in copper-restriction conditions (i.e., addition of BCS to 50 μM) (lane denoted cDNA). PCR was also conducted on genomic DNA, and on DNAse-treated RNA without reverse transcription as positive and negative controls, respectively. The position of the amplicon of the expected size after RT-PCR (red arrow) shows that the presence of transcripts in IGR, indicating that *bp2923* and *bfrG* are part of the same operon. Download FIG S1, TIF file, 0.6 MB. Copyright © 2022 Roy et al. 2022 Roy et al. https://creativecommons.org/licenses/by/4.0/ This content is distributed under the terms of the Creative Commons Attribution 4.0 International license.

To identify transcription start site(s) (TSS) in the *bp2923-2921* locus, we performed 5′ Rapid Amplification of cDNA Ends (5′ RACE) experiments. As the high (G+C) content of *bp2923* made it intractable for this technique, we introduced silent mutations to match its codon usage with that of B. pertussis and inserted the modified gene in the chromosome by allelic exchange, yielding the recombinant strain *BP2923*-OCU (Optimized Codon Usage). 5’RACE analyses of the locus conducted in *BP2923*-OCU identified a TSS 45 bp before the potential initiation codon of *bp2923* (Fig. S2). No additional TSS was identified between *bp2923* and *bfrG*, consistent with RT-PCR results showing transcripts that straddle the *bp2923-bfrG* intergenic region (Fig. S1).

10.1128/mbio.00912-22.6 FIG S2 Determination of the operon transcription start site (TSS). 5’RACE experiments were conducted using successively a primer located in *bp2923* and a nested primer starting directly upstream of the *bp2923* start codon. The screen shot shows the reads from the sequencing reaction. The arrow indicates the TSS. The sense of transcription is from right to left. Download FIG S2, TIF file, 2.9 MB. Copyright © 2022 Roy et al. 2022 Roy et al. https://creativecommons.org/licenses/by/4.0/ This content is distributed under the terms of the Creative Commons Attribution 4.0 International license.

**FIG 2:**
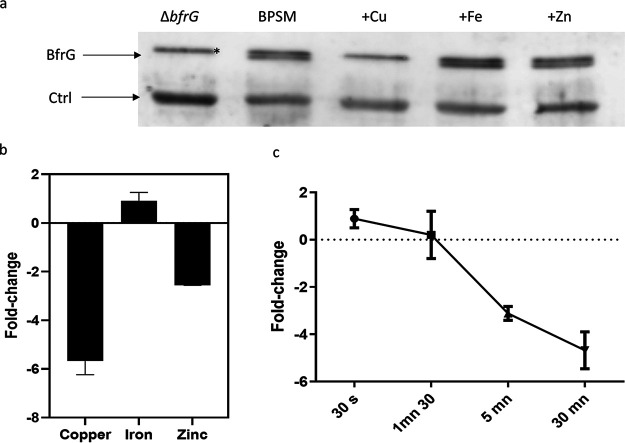
Specificity of the regulation of *bfrG* by metals and kinetics of regulation. a, Analysis of B. pertussis extracts by immunoblotting using anti-BfrG antibodies. *ΔbfrG* represents the deletion mutant. The parental strain BPSM (WT) was used for the last four lanes. The bacteria were grown in standard medium (lanes 1 and 2) or in medium supplemented with 100 μM CuSO_4_ (lane 3), FeSO_4_ (lane 4) or ZnSO_4_ (lane 5). A nonspecific band corresponding to an unidentified protein was used as a loading control. The asterisk indicates another protein just above BfrG which is also recognized by the antibodies. b, Ratios of transcription (in log_2_) of the three genes in bacteria grown in medium supplemented as in (a) relative to bacteria grown in standard medium. Data represent the means of three biological replicates, and the error bars show the SD. c, Kinetics of *bfrG* regulation by copper. Aliquots of BPSM cultures were taken at the indicated times after addition of 100 μM CuSO_4_ to the medium for qRT-PCR analyses on *bfrG*. Data were normalized using a housekeeping gene, and they are compared to the expression level of *bfrG* immediately before the addition of CuSO_4_. The data represent the means of three biological replicates, and the error bars show the SD.

BfrG was detected by immunoblotting analyses in cellular extracts of B. pertussis grown in the absence but not in the presence of copper, whereas Fe and Zn had little effect on its expression, indicating that the regulation of the operon is copper specific (Fig. 2a). Those results were confirmed by qRT-PCR experiments showing a dramatic reduction of *bfrG* mRNA abundance in the presence of Cu (Fig. 2b). In contrast, Fe had no effect and Zn moderately affected *bfrG* mRNA levels, suggesting limited cross regulation.

We performed qRT-PCR experiments on each of the three genes of the operon and normalized the results against a housekeeping gene. We also generated chromosomal reporter fusions by inserting *lacZ* in frame with the first codons of each gene to assess the effect of copper on their expression. *bfrG* and *bp2921* were expressed at moderate levels in copper-restricted medium (Fig. 1c and d). Addition of copper to the medium abolished their transcription and translation, consistent with RNAseq data (24). Intriguingly, *bp2923* was hardly translated, despite being the first gene of the operon, and it did not appear to be regulated by copper (Fig. 1c and d). qRT-PCR analyses on *bfrG* at various times after Cu addition showed a fast decrease in transcript abundance (Fig. 2c).

**FIG 3:**
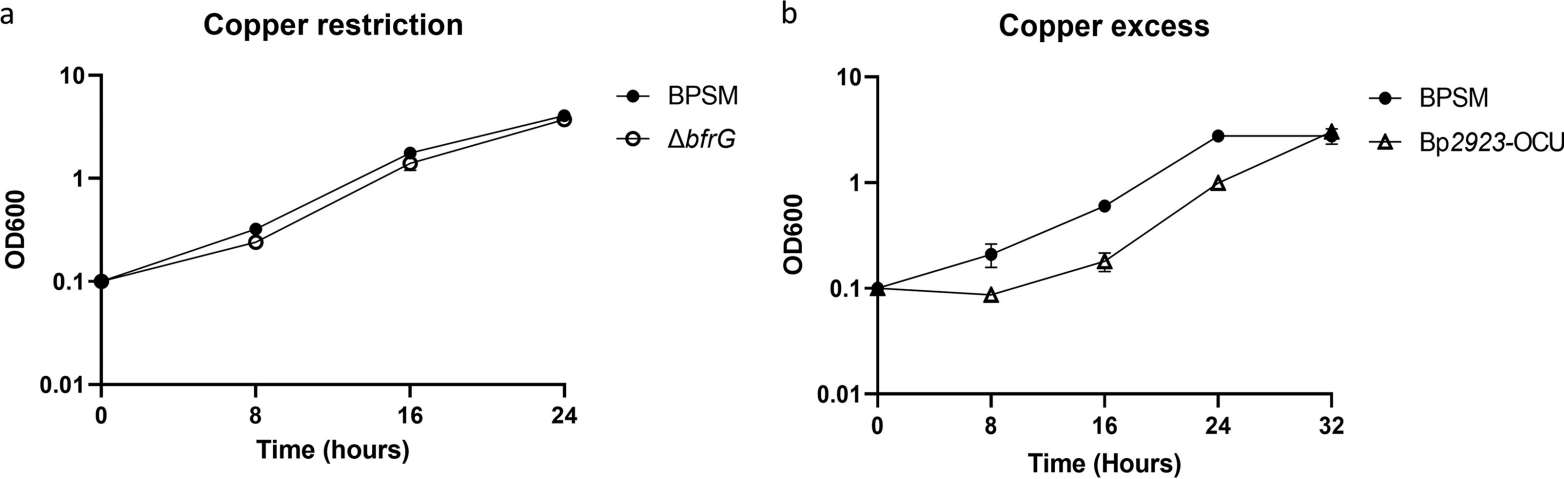
Role of the operon in Cu acquisition. a, Growth of BPSM and BP*ΔbfrG* in Cu-restricted conditions (addition of the copper chelator BCS at 50 μM to the growth medium). b, Growth of BPSM and BP2923-OCU in Cu excess (2 mM CuSO_4_ added to the medium). Note that in BP2923-OCU the expression of the operon poorly responds to copper (see Fig. 4). The latter strain reproducibly showed a growth delay relative to its wt parent, which nevertheless was not significant according to a Mann-Whitney test.

The regulation of this operon indicates that it might mediate Cu acquisition in Cu-restricted conditions. This was tested by comparing the growth of the deletion strain BPΔ*bfrG* with that of its wild-type (wt) parent BPSM, in the presence of the copper chelator bathocuproine disulfonate (BCS) to try and starve the bacteria of copper (Fig. 3a). As the mutant strain displayed no marked growth phenotype under these conditions, we turned to *BP2923*-OCU, which expresses *bfrG* and *bp2921* constitutively (see below), and we compared its growth with that of BPSM in the presence of Cu or BCS. The unregulated expression of the operon delayed bacterial growth in Cu excess (Fig. 3b), even though the differences were not significant. It may be that we have not yet identified the conditions in which this operon plays a role for B. pertussis. Nevertheless, the growth delay of the strain unable to regulate the expression of the operon in response to Cu suggests its function in Cu acquisition and the need for its negative regulation in conditions of Cu excess.

### Importance of *bp2923* for posttranscriptional regulation of *bfrG* and *bp2921*.

**FIG 4:**
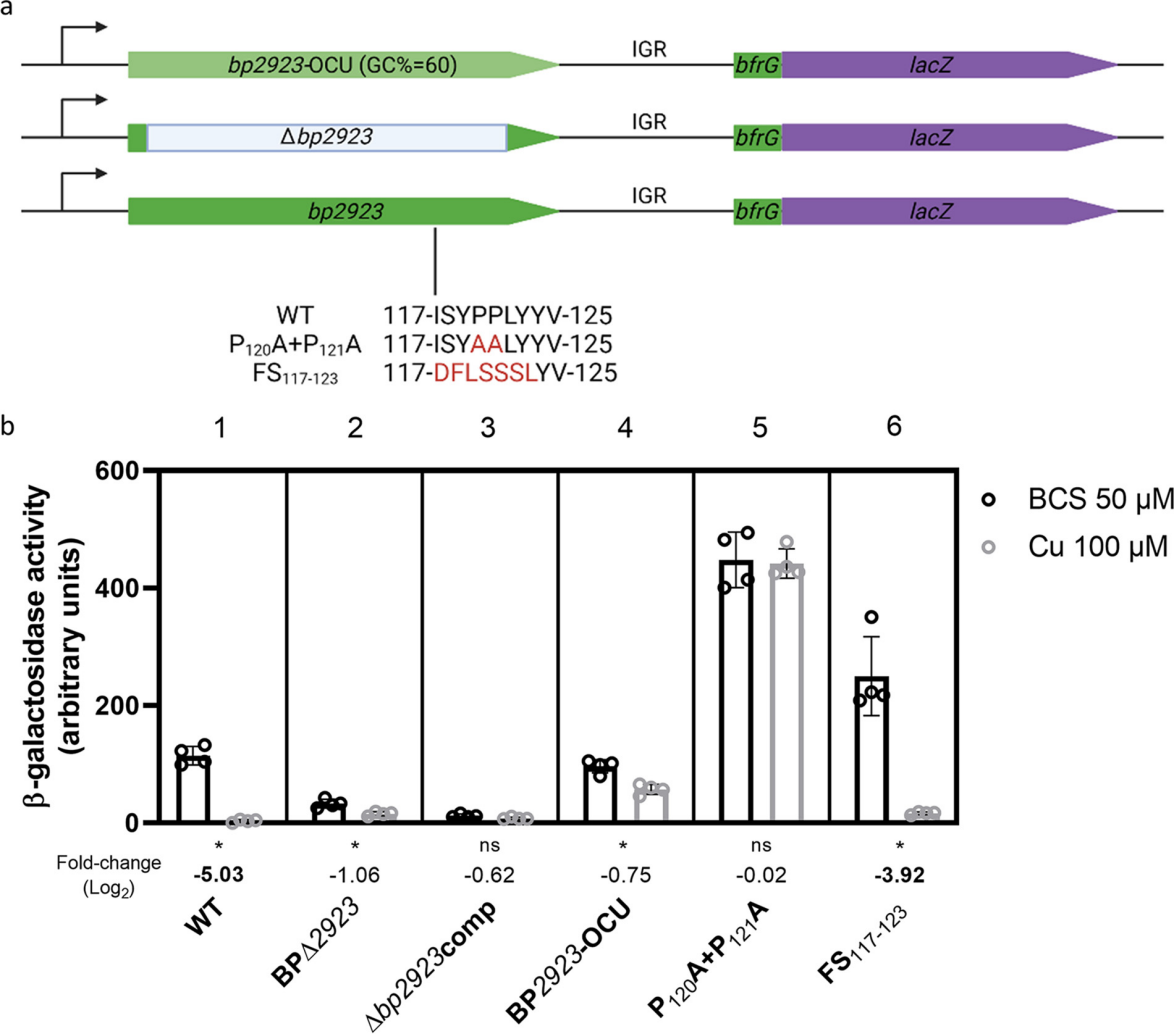
Effects of modifications of *bp2923* on *bfrG* expression. a, Mutations introduced in *bp2923. bp2923*-OCU represents the *bp2923* variant whose codon usage was optimized for B. pertussis. The deletion of *bp2923* (in gray) consists in an in-frame fusion between the first few and last few codons of the gene to avoid polar effects. P_120_A+P_121_A denotes the substitutions of two rare Pro codons by Ala codons, and FS_117-123_ indicates a frameshift obtained by introducing one nucleotide before codon 117 and removing one after codon 123. The resulting amino acid sequences are shown in red. The *bp2923* variants were introduced in the chromosome of *BPΔ2923* by allelic exchange. b, β-galactosidase activities of strains harboring the chromosomal *bfrG-lacZ* fusion in various *bp2923* backgrounds. *Δbp2923*comp represents the complementation of BPΔ2923 by *bp2923* under the control of its own promoter at a distinct chromosomal locus. The individual data points are shown, with the bars representing the means of four biological replicates and the error bars the SD. Statistics were performed using a Mann-Whitney test (*, *P* < 0.05; ns, not significant). As nonparametric tests can sometimes mask reproducible differences, the expression ratios (in log_2_) of the reporter in bacteria grown for 16 h in medium supplemented with 100 μM CuSO_4_ relative to bacteria grown in medium supplemented with 50 μM BCS were also calculated. Thus, one clearly sees that BP2923-OCU responded poorly to Cu (fold change < 2) relative to the wt strain (fold change > 32). Boldfaced letters indicate differences of 4-fold or more between the two conditions.

We investigated a potential regulatory role of the 5′ region of the operon by introducing a large in-frame chromosomal deletion of *bp2923* to avoid polar effects, yielding the recombinant BPΔ*2923* strain (Fig. 4a). We then tested the effect of copper on the expression of *bfrG* using the translational *bfrG-lacZ* fusion. *bfrG* was expressed at very low levels in BPΔ*2923* compared with the parental strain BPSM, and copper regulation was much less pronounced (WT; Fig. 4b, parts 1 and 2). The introduction of *bp2923* at another chromosomal locus in BPΔ*2923* (Δ*2923*comp) did not complement the deletion (Fig. 4b, part 3). Thus, its first position in the operon is required to control both the levels of expression and the regulation by copper of the downstream genes. To determine if the Bp2923 protein or the *bp2923* mRNA was involved in this regulation, we tested the *bfrG-lacZ* fusion in BP*2923*-OCU, in which the protein sequence is intact but the mRNA sequence is modified (Fig. 4b, part 4). *bfrG* was expressed, although at slightly lower levels than in BPSM, and its expression responded poorly to Cu compared with the wt gene, suggesting a role of the mRNA sequence in regulation (Fig. 4b, part 4).

Prediction of the mRNA structure of wt *bp2923* with MFold (http://www.unafold.org/mfold/applications/rna-folding-form-v2.php) indicated very stable potential stem-loop structures in that region (Fig. S3). Strikingly, however, an unstructured 29-bp sequence was predicted in the second moiety of the gene, with a skewed nucleotide content rich in C and T, resulting in several rare codons for B. pertussis. To alter the amino acid sequence of the unstructured mRNA region with minimal perturbation of the mRNA sequence and structure, we introduced reciprocal frameshift mutations (i.e., a frameshift mutation at the beginning of the target sequence followed by a frameshift mutation downstream of that sequence to restore the correct reading frame of the rest of the protein; mutant FS_117-123_, Fig. 4a and Fig. S3). We also replaced two rare CCT (Pro) codons at positions 120 and 121 with frequent GCC (Ala) codons. The P_120_A+P_121_A mutations, which affect the mRNA structure in this region, caused overexpression of the *bfrG-lacZ* fusion and abolished its regulation by copper, unlike the FS_117-123_ mutations (Fig. 4a and b, parts 5 and 6; Fig. S3). Thus, the amino acid sequence encoded in this region appears to be unimportant, whereas mutations that generate secondary structures in the mRNA affected Cu regulation. The lack of structure in this mRNA stretch appears to contribute to the posttranscriptional regulation of the downstream genes.

10.1128/mbio.00912-22.7 FIG S3 Predictions of the mRNA structure of *bp2923* and variants generated in this work. The wt sequence and the sequences of mutants were analyzed between the region coding for the CXXC motif and the STOP codon of *bp2923* using the mfold server (http://www.unafold.org/mfold/applications/rna-folding-form-v2.php). Download FIG S3, TIF file, 0.5 MB. Copyright © 2022 Roy et al. 2022 Roy et al. https://creativecommons.org/licenses/by/4.0/ This content is distributed under the terms of the Creative Commons Attribution 4.0 International license.

### Rho-dependent transcription termination of the operon.

**FIG 5:**
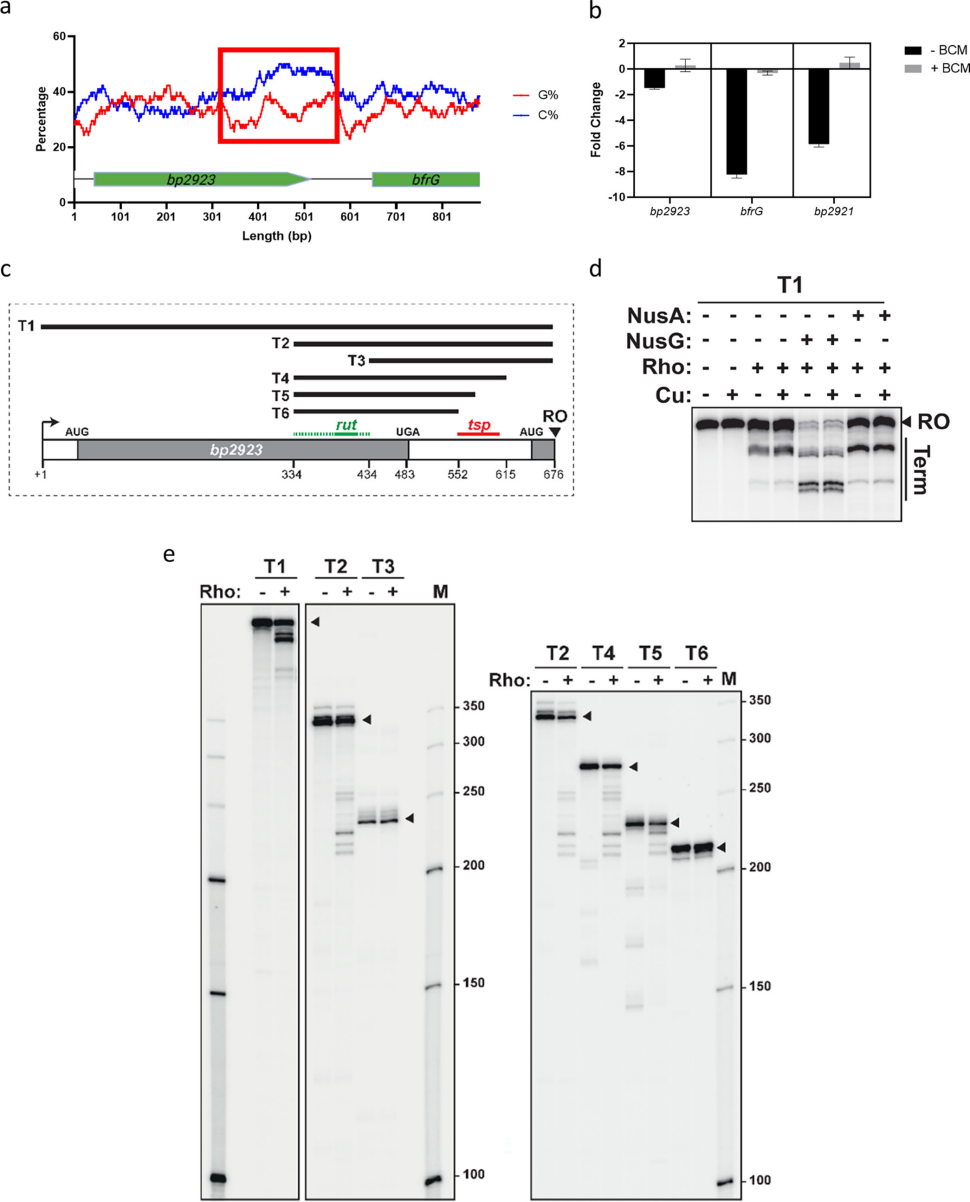
Rho-dependent termination between *bp2923* and *bfrG*. a, Percentages of C and G in the coding strand using a sliding window of 78 nucleotides from the TSS of *bp2923* to the beginning of *bfrG*. The C > G bubble is indicated with a red square. b, qRT-PCR analyses showing the ratios of transcription (in log_2_) of the three genes in bacteria grown in medium supplemented with 100 μM CuSO_4_ relative to bacteria grown in Cu-restricted medium (BCS added to 50 μM), with or without a 30-min treatment with bicyclomycin (BCM). The bars represent the means of three biological replicates and the error bars show the SD. c, Representation of the DNA templates used in the *in vitro* transcription experiments. The regions of the Rho-utilization site (rut) and of the transcription stop point (tsp) identified by the analyses shown in (e) are indicated. d, *In vitro* transcription experiments conducted on the T1 template show the presence of a Rho-dependent terminator in the *bp2923*-IGR region, the enhancement of transcription termination by NusG and NusA and the absence of effect of Cu on termination. RO denotes the runoff product. e, *In vitro* transcription experiments were conducted on all DNA templates to determine the rut and the tsp regions. Arrowheads indicate the RO products.

Posttranscriptional regulation in bacteria may be mediated through transcription attenuation, which occurs by intrinsic or Rho-dependent mechanisms involving distinct mRNA signatures (30). The bacterial motor protein Rho is widely used to control expression of metabolic or stress response genes (31). Rho-utilization (rut) sites are unstructured mRNA regions to which Rho can bind, composed of repeated C-rich patterns (32, 33). Analyses of the nucleotide sequence of the locus revealed a so-called C > G bubble, i.e., a region where the percentage of C is higher than that of G on the coding strand, starting in the unstructured region of *bp2923*, and ending in IGR (Fig. 5a). As C > G bubbles are indicative of Rho-dependent terminators, we analyzed the effect of copper on cultures treated with the Rho-specific inhibitor bicyclomycin using qRT-PCR. This treatment abolished the downregulation of *bfrG* and *bp2921* by copper but had little effect on *bp2923* expression (Fig. 5b). This indicated that the presence of Cu triggers Rho-dependent transcription termination within the *bp2923-bfrG-bp2921* operon between the first two genes (Fig. 5b).

Rho-dependent transcription termination was confirmed by *in vitro* transcription experiments with a DNA template encompassing the sequence from the TSS upstream of *bp2923* to the first nucleotides of *bfrG*. Addition of Rho to the transcription reaction resulted in premature termination that was enhanced by the presence of factors known to facilitate Rho-dependent termination, NusA and NusG (30, 31) (Fig. 5c and d). Copper did not affect transcription termination *in vitro* under these conditions, arguing that the mRNA does not sense Cu by itself, as would be expected if it contained a riboswitch. By using DNA templates truncated from the 5′ or 3′ end, we mapped the transcription stop point (tsp) region in IGR and identified a putative rut site in *bp2923* starting in the C > G bubble region (Fig. 5c and e).

A Rho-dependent termination mechanism can account for the effects of the mutations in the unstructured region (Fig. 4b, parts 5 and 6). Thus, the frameshift mutations did not affect regulation by copper because the rut site was preserved. In contrast, mutations that disrupt the rut site (P_120_A+P_121_A) abolished regulation. The observation that the latter also increased the expression level of *bfrG* in the absence of Cu suggests a background level of termination in the wt operon.

### Importance of Bp2923 protein for *bfrG* expression and regulation.

**FIG 6:**
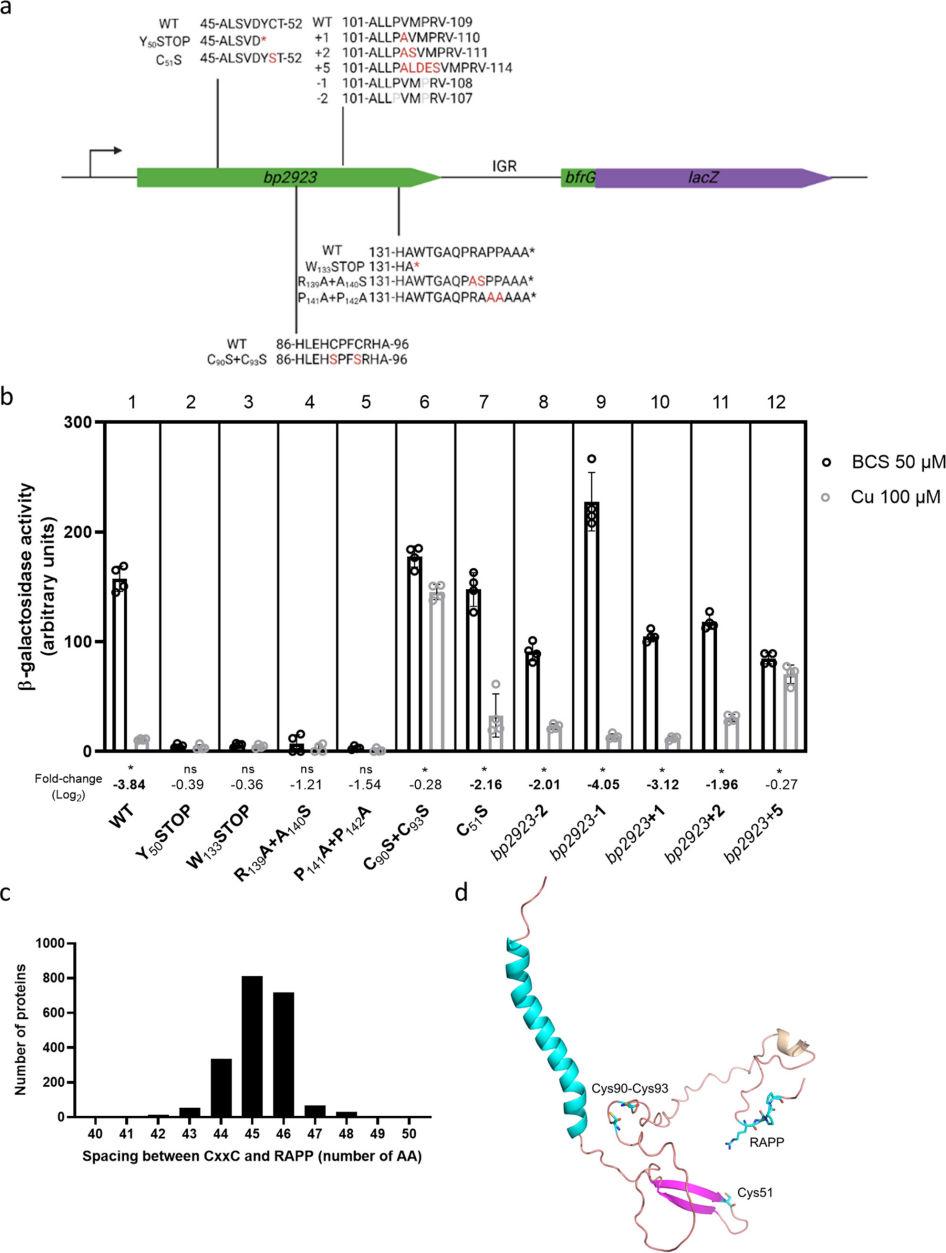
Role of conserved features of the Bp2923 protein for expression and Cu regulation of *bfrG*. a, Chromosomal mutations introduced in *bp2923*. The wt sequence in the region of interest is shown in first position in all cases. The point mutations, the nonsense mutations (denoted with *) or the insertions are indicated in red, and the deletions are in pale gray. We designed these insertions and deletions in such a way as to minimize their impact on the mRNA structure (Fig. S3). b, β-galactosidase activities of strains harboring a chromosomal *bfrG-lacZ* fusion in various *bp2923* backgrounds. The individual data points are shown, with the bars representing the means of four biological replicates, and the error bars the SD. Statistics were performed using a Mann-Whitney test (*, *P* < 0.05; ns, not significant). As nonparametric tests can sometimes mask reproducible differences, the expression ratios (in log_2_) of the reporter in bacteria grown for 16 h in medium supplemented with 100 μM CuSO_4_ relative to bacteria grown in medium supplemented with 50 μM BCS were also calculated. Thus, one clearly sees that the C_90_S+C_93_S and *bp2923 *+ 5 mutants responded poorly to Cu (fold change < 2) relative to the wt strain (fold change ~16). Boldfaced letters indicate differences of 4-fold or more between the two conditions. c, Conservation of the spacing between the CxxC and the RAPP motifs in >2000 DUF2946-family proteins. d, Predicted structure of the Bp2923 protein. The signal-peptide and the conserved residues are shown in cyan. The residues of interest are represented in sticks. The prediction was conducted with AlphaFold2 (https://colab.research.google.com/github/sokrypton/ColabFold/blob/main/AlphaFold2.ipynb).

We next investigated a potential role of the *bp2923*-encoded protein for regulation. We introduced nonsense codons at positions 50 or 133 to cause premature translation termination with minimal disruption of the mRNA sequence and structure (mutants Y_50_STOP and Y_133_STOP; Fig. 6a). Both mutations abrogated reporter activity of the *bfrG-lacZ* fusion (Fig. 6b, parts 2 and 3), showing that premature release of the ribosome abolishes expression of the downstream gene even in the absence of Cu, probably because the lead ribosome limits Rho access to the rut site and/or to the RNA polymerase. The observation that translation of *bp2923* is required for expression and regulation of the downstream genes indicates that this gene represents a new type of regulatory upstream ORF (uORF) (34).

### Role of conserved features of the Bp2923 protein for regulation.

The Bp2923 protein belongs to the DUF2946 family, which is characterized by two highly conserved sequence motifs. The RAPP motif, which is encoded by frequent B. pertussis codons, is located at positions 139 to 142, three residues before the C terminus (Fig. 1b). This motif is reminiscent of the C-terminal RAGP sequence of the so-called arrest peptide of a well-known uORF that regulates *secA* expression in Escherichia coli, SecM (35). Arrest peptides cause ribosome stalling by interacting with the ribosome tunnel, which positively or negatively affects the expression of downstream genes (35). We thus tested the importance of the C-proximal RAPP motif by replacing Arg_139_ and Ala_140_ with Ala and Ser, or the two Pro with Ala residues (mutants R_139_A+A_140_S and P_141_A+P_142_A, respectively; Fig. 6a). Both sets of modifications abolished *bfrG* expression even in the absence of copper (Fig. 6b, parts 4 and 5). This “constitutive” termination of transcription shows that conversely, the RAPP sequence is required to promote transcription of the rest of the operon in copper-restricted conditions. Therefore, the conserved RAPP motif is most likely part of a ribosome arrest peptide involved in the regulation process. In enterobacteria, slow translation of consecutive Pro residues is alleviated by a specific elongation factor, EF-P (36). However, a knockout mutation of this gene in B. pertussis had no effect on the expression of *bfrG* or its regulation by Cu (Fig. S4). Thus, EF-P is not involved in relieving ribosome stalling in the presence of copper.

10.1128/mbio.00912-22.8 FIG S4 Effect of an EF-P knock-out on *bfrG* regulation. Left panel, qRT-PCR analyses showing the ratios of transcription of *bfrG* in bacteria grown for 16 h in medium supplemented with 100 μM CuSO_4_ relative to bacteria grown in Cu-restricted medium (BCS added to 50 μM). The results (in log_2_) were normalized against a housekeeping gene. Right panel, analysis of B. pertussis extracts by immunoblotting using anti-BfrG antibodies. The bacteria were grown for 16 h in SS medium supplemented with 100 μM Cu or 50 μM BCS. A nonspecific band corresponding to an unidentified protein band was used as a loading control. Download FIG S4, TIF file, 0.4 MB. Copyright © 2022 Roy et al. 2022 Roy et al. https://creativecommons.org/licenses/by/4.0/ This content is distributed under the terms of the Creative Commons Attribution 4.0 International license.

The other hallmark motif in the DUF2946 protein family, CXXC at positions 90 to 93, is a recognized Cu-binding motif. We replaced the two Cys residues with two Ser residues and determined the effect of the SxxS sequence on regulation of *bfrG-lacZ* by copper (mutant C_90_S+C_93_S; Fig. 6a). These modifications did not affect the expression level of *bfrG* but strongly impaired its control by copper, demonstrating the involvement of the CXXC motif in regulation (Fig. 6b, part 6). In contrast, replacement of a semiconserved Cys in the DUF2946 family preserved the regulation of *bfrG* (mutant C_51_S; Fig. 6b, part 7).

The CXXC and the RAPP motifs are separated from each other by 45 intervening residues. Genome mining identified more than 2000 DUF2946 protein sequences in databases, mostly in Proteobacteria (Table S1), and their analysis showed that the spacing between the two motifs is conserved to within one or two residues in the family (Fig. 6c). We therefore probed its importance for expression and regulation of *bfrG* by shortening or lengthening the spacing by one, two, or five residues in the Bp2923 protein (mutants *bp2923-1*, *bp2923-2*, *bp2923 + 1*, *bp2923 + 2* and *bp2923 + 5*, respectively; Fig. 6a). We designed those mutations to minimize their effects on the mRNA structure (Fig. S3). Deletion or addition of one or two residues moderately affected the *bfrG* expression levels, but not its regulation by copper. In contrast, addition of five residues strongly reduced the effect of copper, showing that the two motifs must be adequately spaced for proper regulation, within a limited degree of variation (Fig. 6b, parts 8 to 12).

10.1128/mbio.00912-22.2 TABLE S1 List of CruR homologs and taxonomy of the host organisms. Download Table S1, DOCX file, 0.3 MB. Copyright © 2022 Roy et al. 2022 Roy et al. https://creativecommons.org/licenses/by/4.0/ This content is distributed under the terms of the Creative Commons Attribution 4.0 International license.

*In silico* analyses predicted the presence of an export signal, i.e., a signal-peptide or an N-terminal transmembrane segment with an N_in_-C_out_ orientation in more than 95% of all DUF2946 proteins. As the signal-peptide of SecM has been implicated in posttranscriptional regulation of the downstream *secA* gene (35, 37), we tested the possibility that the N-terminal region of Bp2923 similarly participates in Cu regulation. We thus introduced reciprocal frameshift mutations to replace 40 residues encompassing the putative export signal by an out-of-frame sequence devoid of signal-peptide features while keeping the natural sequence of the rest of the protein (mutant FS_5-44_; Fig. S5). This modification did not affect the expression or the regulation of *bfrG*.

10.1128/mbio.00912-22.8 FIG S5 Replacement of the predicted signal-peptide of Bp2923 by reciprocal frameshift mutations. a, WT and mutant protein sequences in that region. b, β-galactosidase activities of strains harboring the chromosomal *bfrG-lacZ* fusion in the indicated *bp2923* backgrounds. The individual data points are shown, with the bars representing the means of four biological replicates, and the error bars the SD. Statistics were performed using a Mann-Whitney test (*, *P* < 0.05; ns, not significant). The expression ratios (in log_2_) of the reporter in bacteria grown for 16 h in medium supplemented with 100 μM CuSO_4_ relative to bacteria grown in medium supplemented with 50 μM BCS were also calculated. Download FIG S5, TIF file, 0.4 MB. Copyright © 2022 Roy et al. 2022 Roy et al. https://creativecommons.org/licenses/by/4.0/ This content is distributed under the terms of the Creative Commons Attribution 4.0 International license.

### Genes in synteny with *bp2923* homologs.

**FIG 7:**
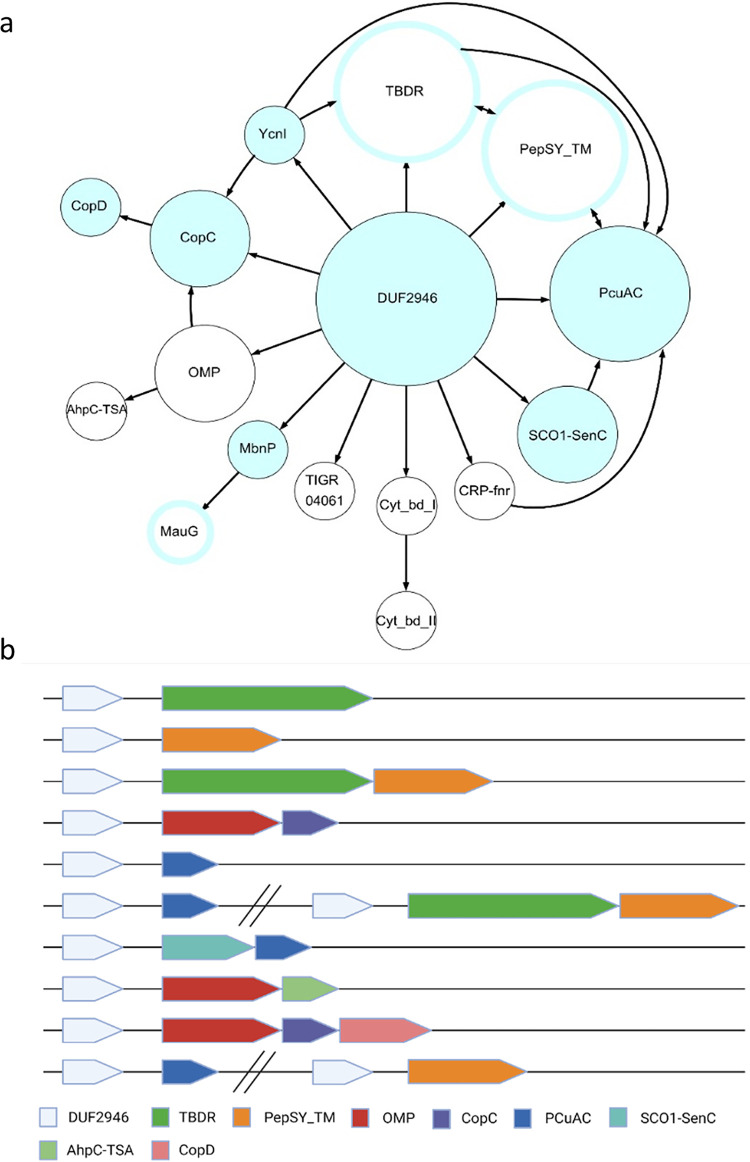
Genetic environments of *cruR* homologs. a, DUF2946-coding genes were systematically found in first positions of putative operons that comprise genes coding for proteins of the indicated families, represented by circles whose sizes correspond to the current numbers of occurrences of each protein type in those operons (large circle, 400 to 1000; medium-size, 50 to 399; small circles, 1 to 49). Blue-filled circles represent protein families involved in copper homeostasis or utilization, and those surrounded by blue lines represent families some members of which are found in copper-related operons, while others may be involved in distinct processes. The arrows indicate the order of the genes in the putative operons. b, Representation of the most frequent genetic organizations. Parallel bars separate potentially distinct transcriptional units at the same locus.

*In silico* analyses revealed that Bp2923 homologs are widespread among β, γ, and alphaproteobacteria (Table S1). A few were also found in Planctomycetes, Firmicutes, and Deinococcus. In most cases, potential operonic structures were identified with these genes in first position (Fig. 7a and b). *bp2923* homologs are often found to precede TBDT- and/or PepSY_TM-coding genes, as in B. pertussis, indicating a widespread genetic organization in bacteria. Among those TBDTs are OprC-type transporters (signature TIGR01778), one of which was recently shown to bind Cu (38). Other genes frequently found in operons with *bp2923* homologs code for putative proteins involved in Cu transport, binding, or homeostasis, including the copper chaperones ScoC and Pcu_A_C that participate in the assembly of heme-copper subunits of respiratory complexes (39, 40), and the Cu-binding proteins YcnI and CopC (41, 42). These operons also comprise MbnPH-like genes notably found in biosynthesis operons of copper-binding methanobactin-type molecules (43, 44) and AhpC_TSA genes, whose products detoxify peroxides (45). Long intergenic distances between *bp2923* homologs and the following genes are generally observed, indicating a potential role in regulation.

## DISCUSSION

Bacteria have evolved complex mechanisms to respond to changes of their environment, and notably to strictly regulate the availability of necessary but harmful transition metals. Copper controls homeostasis genes through transcriptional regulation (11, 16, 17), whereas other biologically relevant transition metals also regulate gene expression through posttranscriptional mechanisms involving sRNAs or riboswitches (18, 46, 47). In this work, we unveiled a mechanism of posttranscriptional control of a Cu acquisition system, involving an uORF that codes for a protein of the DUF2946 family. We propose to name this new regulatory protein CruR for Copper-responsive upstream Regulator.

The bacterial motor protein Rho is a general attenuator of transcription widely used to control expression of metabolic or stress response genes (31). Evidences suggesting that regulation of the operon proceeds via Rho-dependent termination are the loss of responsiveness of the system to copper *in vivo* in the presence of the Rho-specific inhibitor bicyclomycin and the identification of a Rho-dependent terminator *in vitro*. Rho-utilization sites are found in unstructured mRNA regions composed of repeated, C-rich patterns (33). Accordingly, *in vitro* transcription experiments coarsely mapped a rut site to the unstructured C > G bubble region in *cruR.*

**FIG 8:**
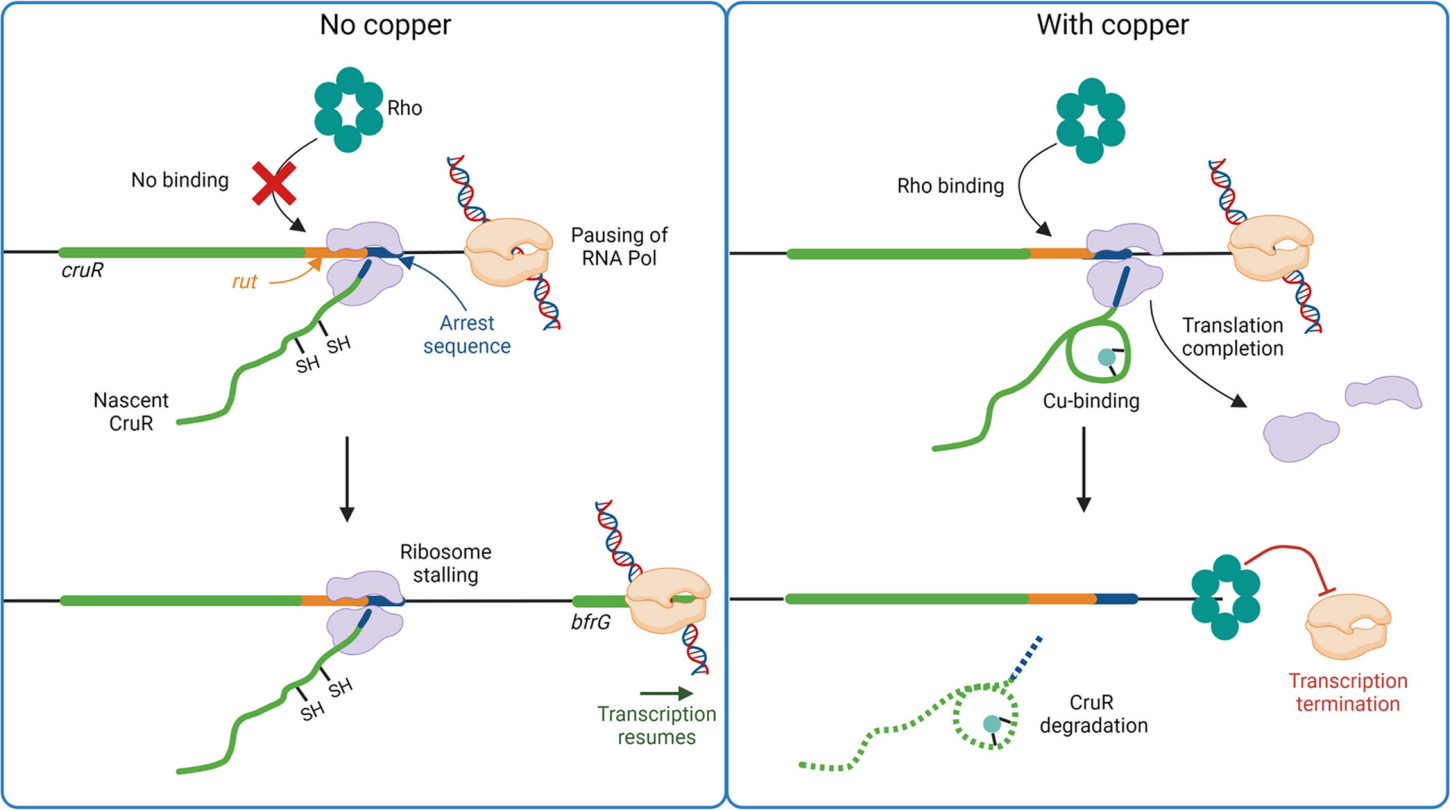
Model of *bfrG* regulation by the uORF CruR. The RNA polymerase pauses in the intergenic region between *cruR* and *bfrG*, and the lead ribosome translating CruR stalls at the RAPP motif, which is part of an arrest peptide (shown in dark blue). In the absence of copper (left panel), ribosome occupancy on this motif prevents Rho from binding to the rut site (in orange) on the mRNA or from accessing the RNAP polymerase, and transcription resumes, followed by translation of the rest of the operon. The perception of Cu (right panel) by nascent CruR through its CXXC motif relieves stalling of the ribosome, which completes the translation of CruR. Rho can access the rut site and the RNA polymerase, leading to transcription termination. CruR is most likely exported and degraded.

The following model of a ligand-dependent relief of translation arrest is consistent with all our data (Fig. 8). Following transcription of *bp2923*, the RNA polymerase most likely pauses in the intergenic region. Transcriptional pausing notably facilitates interactions with regulatory proteins, and termination stop points often coincide with pausing sites (48). In the absence of copper, stalling of the lead ribosome at the conserved RAPP motif of nascent CruR prevents Rho from binding to the Rut site and contacting the RNA polymerase, which enables the latter to resume transcription of the rest of the operon. The stalled ribosome is presumably rescued by a quality-control mechanism as reported for SecM (49, 50). In the presence of Cu in the cytoplasm, its perception by the invariant CXXC motif of nascent CruR relieves ribosome stalling, leading to completion of CruR translation. The mechanism of stalling relief remains to be deciphered but does not involve the signal peptide of CruR or EF-P, unlike with other uORFs (37, 51, 52). The observation that the spacing between the two conserved motifs plays a role in regulation suggests that Cu binding to the nascent protein triggers some cotranslational folding. Protein folding might exert a force that relieves stalling, as described in other systems (53, 54). Ribosome dissociation from the mRNA enables Rho to contact the RNA polymerase, causing transcription termination before *bfrG.* CruR is the first characterized uORF mediating posttranscriptional regulation in response to a transition metal.

CruR appears to be translated at low levels (Fig. 1), and it was not detected in earlier proteomic analyses (24). It is predicted to be poorly structured (Fig. 6d), and our attempts to produce it as a recombinant protein were unsuccessful. All these observations suggest that mature CruR is short-lived and therefore unlikely to exert an additional function in B. pertussis. CruR is most likely rapidly degraded given that C-terminal nonpolar residues constitute a signal for degradation by various proteases (55, 56). Notably, metal-binding proteins and regulatory proteins are ranked as fast degrading in E. coli (57).

The intergenic region between *cruR* and *bfrG* is 162-bp long and probably highly structured given its high (G+C) content. Long intergenic distances between *cruR* homologs and the following genes are generally observed. Of note, other types of uORFs are also followed by long intergenic regions (58), suggesting that this feature is necessary for regulation. Such distances are incompatible with translational coupling between *cruR* and the following gene, which implies *de novo* translation initiation of the next gene. In the B. pertussis case, this is consistent with the higher levels of activity of the BfrG translational fusion compared with the CruR fusion.

As *cruR* genes are overwhelmingly found in first position of putative operons in synteny with genes linked to Cu homeostasis, and the two functional motifs of the proteins and their spacing are extremely well conserved, *cruR* is most likely the paradigm of a new family of uORF involved in posttranscriptional regulation in response to copper. There are indeed indications that our model of a copper-responsive uORF regulating copper homeostasis genes may apply to other cases. The *oprC* gene in Pseudomonas aeruginosa is downregulated by Cu (59) and preceded by a *cruR* homolog. In Bradyrhizobium japonicum, an operon for cytochrome oxidase biogenesis induced by copper starvation is also headed by a *cruR* homolog (39).

Finally, it is interesting that posttranscriptional regulation by Cu involves an uORF rather than a riboswitch, and that no riboswitches have been described for transition metals at the top of the Irving-Williams series, Cu and Zn. Known metallo-riboswitches have micromolar affinities for their ligands (20, 60). The extremely low levels of free intracellular copper would prevent low-affinity riboswitches from outcompeting high-affinity copper-binding proteins (16), which may be the reason why protein-based posttranscriptional regulation mechanisms have evolved for copper. There are preliminary indications that other families of uORFs might posttranscriptionally regulate genes coding for copper export systems in response to the metal (61–63). In a recent work, the activity of a multicopper oxidase, CutO, was shown to depend on an upstream ORF with similarities to CruR, CutF, which the authors conjectured works as a Cu chaperone (64). It is tempting to speculate that CutF may also be an uORF that activates the expression of its downstream genes in response to copper. Many variations on the theme of Cu-responsive posttranscriptional regulation by uORFs most likely remain to be discovered.

## MATERIALS AND METHODS

### Bacterial strains and culture conditions.

B. pertussis strains were grown on Bordet-Gengou (BG) agar supplemented with 10% sheep blood for 48 hours at 37°C, and then in modified Stainer-Scholte (SS) medium at 37°C with agitation. SS medium was supplemented with 50 μM of bathocuproine disulfonate (BCS) to limit copper availability or with 100 μM CuSO_4_ to supply copper. Where indicated to generate a large excess of copper, CuSO_4_ was added to 2 mM. Antibiotics were added at 100 μg/mL streptomycin, 10 μg/mL gentamycin, 30 μg/mL nalidixic acid, 150 μg/mL ampicillin, and 25 μg/mL kanamycin. Where indicated, cultures were treated with 20 μg/mL bicyclomycin for 30 min at mid-log phase.

### Construction of mutant strains.

Deletion mutants of *cruR* and of *bfrG* were constructed by amplifying their flanking regions as EcoRI-XbaI and XbaI-HindIII fragments and cloning the amplicons in tandem in pSS1129 (65). The *bp2923*-OCU synthetic gene also containing the flanking regions of *cruR* (*bp2923*) was purchased from GeneCust and introduced in pSS1129. Recombinant pSS1129 plasmids were used to transform E. coli SM10 for conjugation with B. pertussis BPSM, to perform allelic exchange. Antibiotic selection was then conducted appropriately to select the recombinant strains. To construct the *cruR-lacZ* fusion, the sequence encompassing *bp2924*, the intergenic *bp2924-cruR* region, and the first 10 codons of *cruR* was amplified using oligonucleotides carrying EcoRI and XhoI sites, respectively, and the amplicon was cloned in pQC2123 (66) digested with EcoRI and SalI. For *bfrG-lacZ*, the amplicon included *bp2924*, *cruR*, IGR and the first 10 codons of *bfrG*. For *bp2921-lacZ*, the amplicon included 600 bp upstream of *bp2921* and its first 10 codons. The C_51_S, W_133_STOP and frameshift mutations were introduced by site-directed mutagenesis of *cruR* on pUC57-*cruR* using the kit QuikChange II XL. This plasmid carries a synthetic 800-bp EcoRI-XhoI fragment starting in *bp2924* and ending after the first 10 codons of *bfrG*. The mutated fragments were introduced in pQC2123 as above. Synthetic gene fragments were ordered from GeneCust to introduce the Y_50_STOP, P_120_A+P_121_A, C_90_S+C_93_S, R_139_A+A_140_S, *bp2923+1*, *bp2923-1*, *bp2923+2*, *bp2923-2*, and *bp2923+5* mutations in *cruR.* Using the natural NcoI site in *cruR*, the EcoRI-NcoI or NcoI-XhoI fragments of pUC57-*cruR* were replaced with their mutated counterparts, and the complete EcoRI-XhoI fragment was ligated with pQC2123. All pQC2123 variants were introduced in BPΔ*2923* by conjugation and integrated in its chromosome by using the 600 bp sequence upstream of *cruR* for homologous recombination. For complementation of *cruR* in trans in the BP*Δ2923* chromosome, the BamHI-XbaI fragment of pRM1 was replaced by an amplicon encompassing *cruR* and its promoter region. pRM1 derives from pXR1 (67), from which the HindIII-ApaLI fragment was replaced with a synthetic construct containing a 666-bp HindIII-BamHI portion of *ureJ*, a central 3191-bp BamHI-XbaI portion of *fhaB* and a 1064-bp XbaI-ApaLI portion of *ureC*. *bp2923* was introduced at the inactive *ure* locus of B. pertussis by homologous recombination. A knock-out mutant of *efp* was constructed by interrupting the gene with a recombinant pFUS2 suicide plasmid (68). The plasmids and oligonucleotides are described in Tables S2 and S3.

10.1128/mbio.00912-22.3 TABLE S2 Plasmids used in this study. Download Table S2, DOCX file, 0.02 MB. Copyright © 2022 Roy et al. 2022 Roy et al. https://creativecommons.org/licenses/by/4.0/ This content is distributed under the terms of the Creative Commons Attribution 4.0 International license.

10.1128/mbio.00912-22.4 TABLE S3 Oligonucleotides used in this study. Download Table S3, DOCX file, 0.02 MB. Copyright © 2022 Roy et al. 2022 Roy et al. https://creativecommons.org/licenses/by/4.0/ This content is distributed under the terms of the Creative Commons Attribution 4.0 International license.

### Immunoblot analyses.

The bacterial pellets from 10-mL B. pertussis cultures grown overnight to an OD_600_ of 1 to 1.5 in Cu-restricted or Cu-supplemented media were resuspended to an OD_600_ of 5 in 50 mM Tris-HCl (pH 8) and lysed using a Ribolyser at speed 6 for 50 s. After SDS-PAGE and transfer of the proteins on a nitrocellulose membrane BfrG was detected by immunoblotting using a polyclonal antibody produced in guinea pig (Eurogentec, Belgium) at a 1:2,500 dilution, followed with anti-guinea pig-HRP antibodies at a 1:5,000 dilution. Blots were revealed using the Amersham ECL Prime Western Blotting System with the Amersham Imager 600 (GE). An unidentified protein recognized by the antibodies on the blots was used as a loading control.

### RNA techniques.

8 mL of liquid B. pertussis cultures grown in Cu-restricted or Cu-supplemented media as above were centrifuged at 4000 rpm at 4°C for 10 minutes after adding 2 mL of a 95/5 ethanol/phenol mix. Pellets were flash-frozen in liquid nitrogen and kept at −80°C. For RT-PCR, RNA extraction was performed using Tri-Reagent (InVitrogen), followed by a DNAse I treatment (Sigma Aldrich) to remove remaining genomic DNA. Retro-transcription was performed with the Verso cDNA synthesis kit (ThermoFisher). qRT-PCR was performed in a Roche LightCycler 480 Instrument II using the Takyon LowROX SYBR kit (Eurogentec). All qRT-PCR experiments were conducted with 3 biological replicates and 3 technical replicates, and data were normalized using the housekeeping gene *bp3416*. This gene coding for the purine biosynthesis protein PurH has been repeatedly used for normalization of qRT-PCR, as its expression level is constant. In particular, *bp3416* was expressed at the same levels irrespective of the presence of Cu in the medium (24).

5’RACE experiments were conducted on total RNA extracted from cultures of *BP2923*-OCU supplemented with BCS, using the Generacer kit (Invitrogen) and specific RACE primers for *bfrG* and *cruR* according to the manufacturer’s instructions. For *cruR*, after PCR amplification with a first primer annealing within the gene, a nested PCR was performed using a second primer annealing immediately before the *cruR* start codon to enhance specificity. The cDNA isolated in the RACE experiment was used to build a library using the Illumina TruSeq Stranded RNA LT library preparation kit, followed by sequencing on an Illumina NextSeq 500 benchtop sequencer. The GeneRacer adapter sequence was removed from the reads using Cutadapt (https://github.com/marcelm/cutadapt) and the reads were mapped using the CLC Genomics software (Qiagen).

### β-galactosidase activity measurements.

B. pertussis strains carrying chromosomal translational fusions with *lacZ* were cultured to an OD_600_ of 1.5 to 2 in the indicated conditions and harvested by centrifugation. Pellets were resuspended to an OD_600_ of 5 and lysed using a Ribolyser at speed 6 for 50 s. β-galactosidase activity was measured as described (68). Experiments were conducted with 4 biological replicates and 3 technical replicates.

### Statistics.

Four biological replicates were used in β-galactosidase activity studies. Statistical analyses were performed with the GraphPad Prism software using the nonparametric Mann-Whitney test with a confidence level of 95%. No statistical analyses were performed for qRT-PCR experiments as they were carried out on three independent biological samples only.

### Transcription termination experiments.

DNA templates T1 to T6 which contain distinct parts of the 5’UTR-*cruR-*IGR region were prepared by standard PCR procedures (69). Briefly, recombinant pQC2123 with the wt locus sequence was amplified with pairs of forward and reverse primers (Table S2). Forward primers allow introduction of the sequence of the T7A1 promoter upstream of the probed B. pertussis sequence. Purification of the Rho, NusA and NusG proteins from E. coli was described (69). These proteins were used as proxies for their B. pertussis counterparts to seek Rho-dependent termination sites within the 5’UTR-*bp2323*-IGR region. Standard transcription termination experiments were performed with E. coli RNAP (69) with minor modifications. Briefly, DNA template (0.1 pmol), E. coli RNA polymerase (0.3 pmol; New England Biolabs), Rho (0 or 1.4 pmol hexamers), NusA (0 or 2.8 pmol), NusG (0 or 2.8 pmol), Superase-In (0.5 U/μL; Ambion), and CuCl_2_ (0 or 10 μM, final concentration) were mixed in 18 μL of transcription buffer (40 mM Tris-HCl, pH 8.0, 5 mM MgCl_2_, 1.5 mM DTT, and 100 mM KCl). DTT in the transcription buffer reduces Cu^2+^ to Cu^1+^, the latter being the form found in the bacterial cytoplasm. Mixtures were incubated for 10 min at 37°C before addition of 2 μL of initiation solution (250 μg/mL rifampicin, 2 mM ATP, GTP, and CTP, 0.2 mM UTP, and 2.5 μCi/μL α[^32^P]UTP in transcription buffer). After 20 min of incubation at 37°C, transcription reactions were stopped by the addition of 4 μL of EDTA (0.5 M), 6 μL of tRNA (0.25 mg/mL), and 80 μL of sodium acetate (0.42 M), followed by ethanol precipitation. Reaction pellets were resuspended in loading buffer (95% formamide; 5mM EDTA) and analyzed by denaturing 7% polyacrylamide gel electrophoresis and by phosphorimaging with a Typhoon FLA-9500 instrument and ImageQuant TL software (GE Healthcare). Potential Cu^2+^ scavenging-by-buffer effects (70) were ruled out in control transcription termination experiments where Tris-HCl was replaced by MOPS, pH 7.9 (not shown).

### *In silico* analyses.

The nonredundant NCBI database (release of December 2020) was searched for the occurrence of DUF2946 using its PFAM hmm profile (PF11162) and the hmmsearch program (http://hmmer.org/). The results were curated to retain proteins less than 80% identical in sequence using cd-hit (http://cd-hit.org). Using a locally built database containing all the bacterial GenBank files of NCBI, the genetic environments of all the genes coding for DUF2946 proteins were extracted. Specifically, the five genes flanking the DUF2946 genes on either side were retrieved irrespective of their distance and translated, and the corresponding protein identification numbers were attached. The sense of transcription of the neighboring genes and their intergenic distances were determined. Putative operons harboring genes transcribed in the same direction as the DUF2946-coding gene were retained. The number of occurrences of given Pfam domain-coding genes at each position relative to the gene of interest, itself found at position “0” of each locus, were then computed. The CLC software was used for sequence manipulation and analyses such as Pfam domain prediction.
