# Supplementary figures and images for: Posttranscriptional Regulation by Copper with a New Upstream Open Reading Frame
Source: mBio. 2022 Jul 13;13(4):e00912-22. doi: 10.1128/mbio.00912-22 (PMC9426467; doi:10.1128/mbio.00912-22)

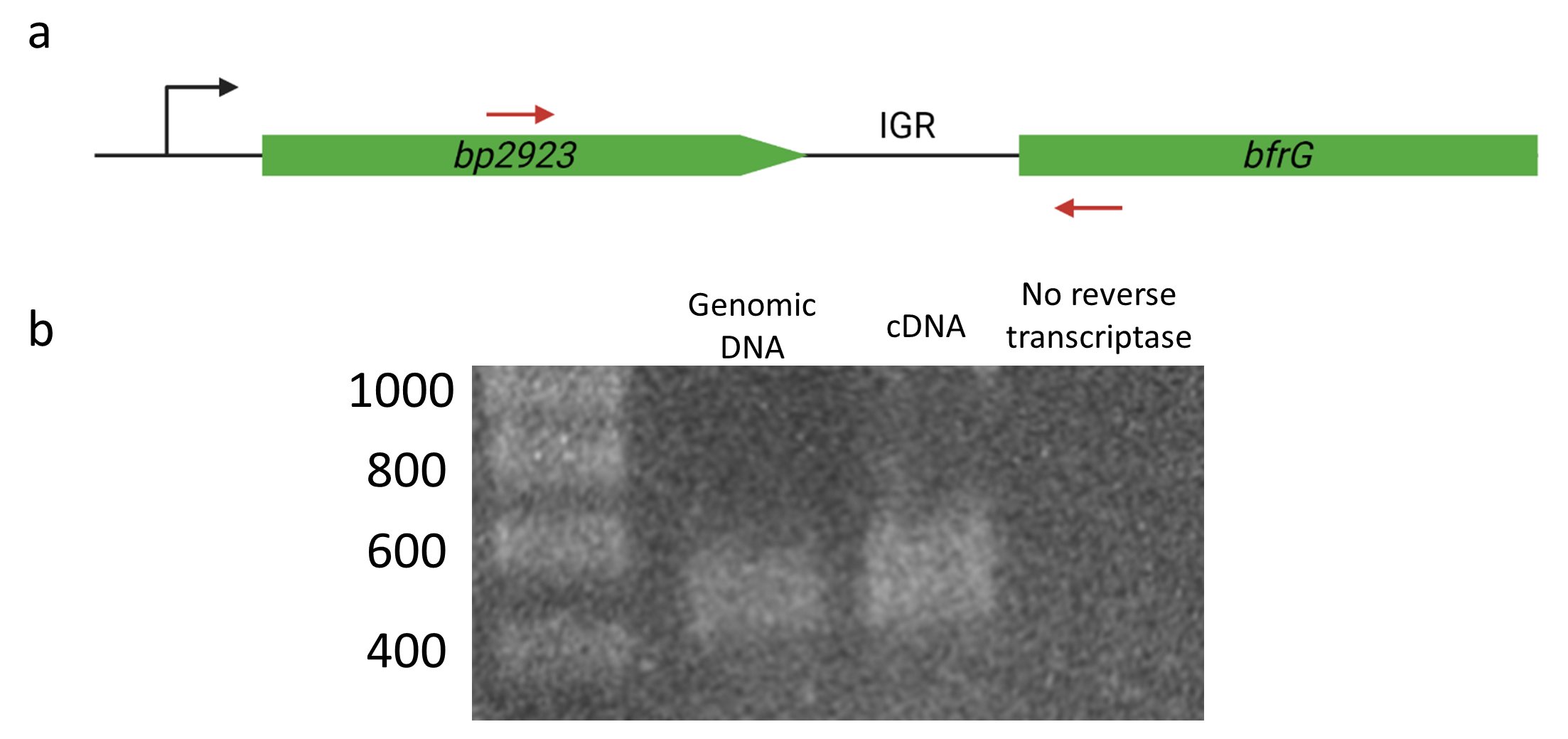

Supplement: FIG S1 [file mbio.00912-22-s0001.tif]

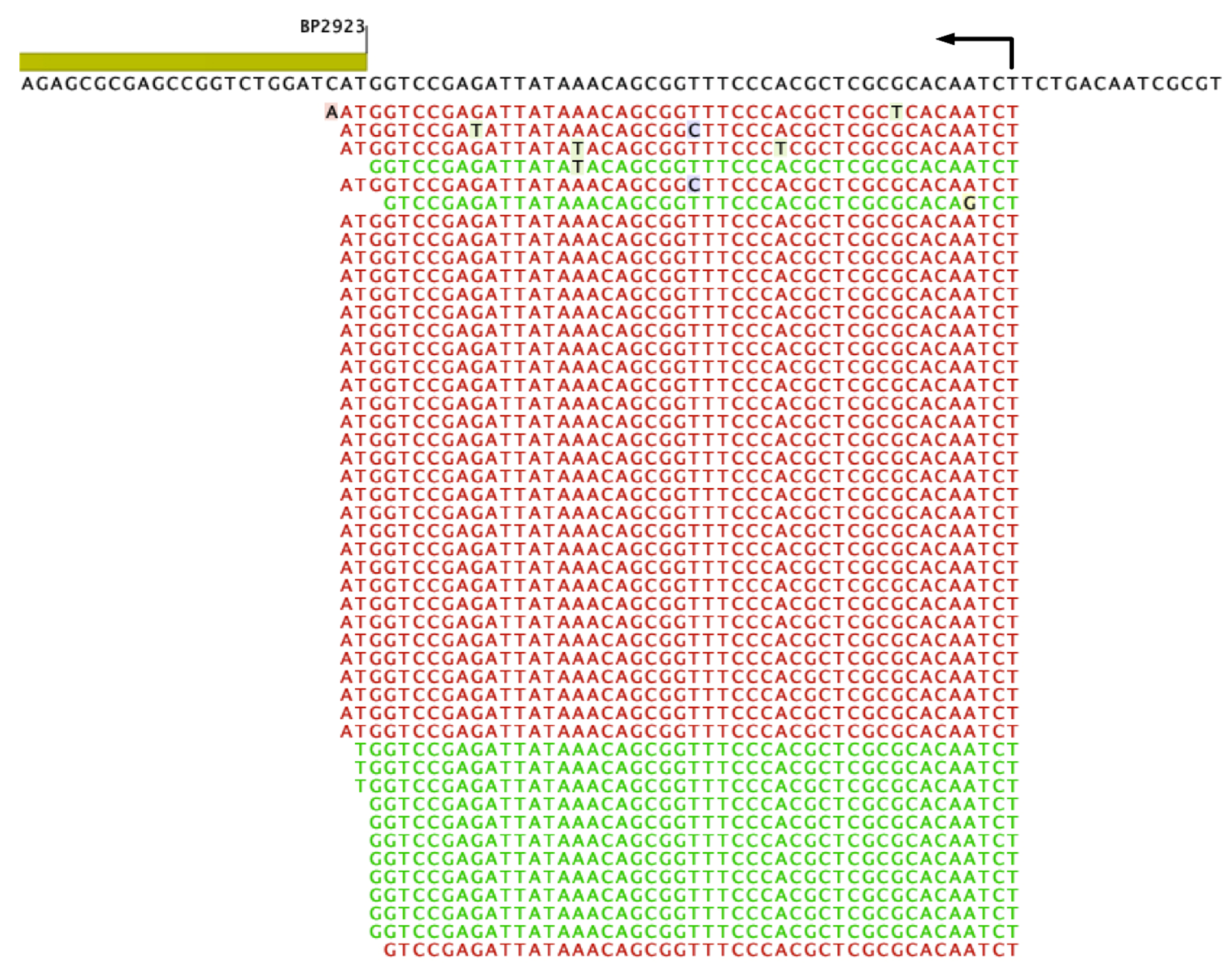

Supplement: FIG S2 [file mbio.00912-22-s0002.tif]

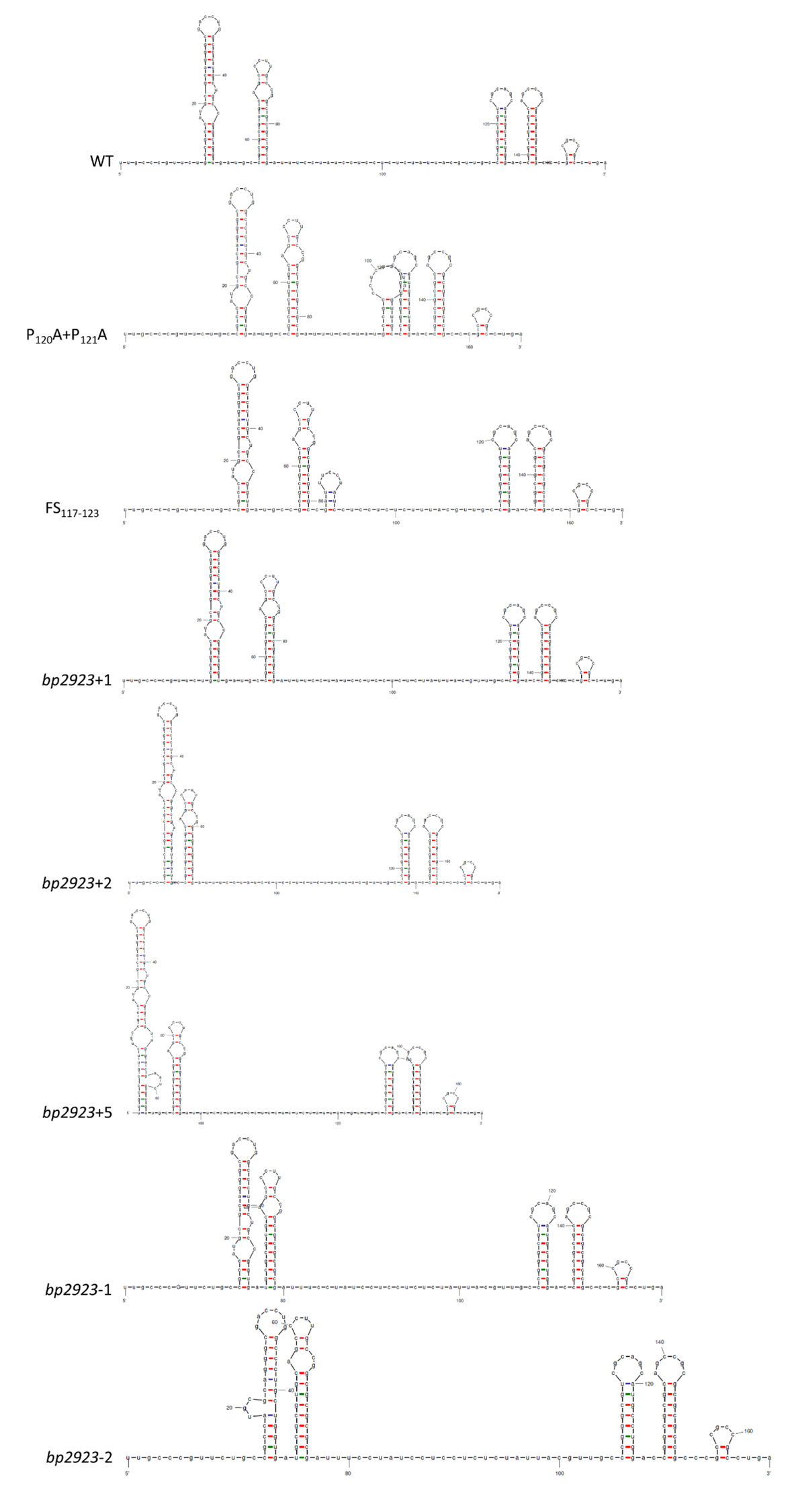

Supplement: FIG S3 [file mbio.00912-22-s0003.tif]

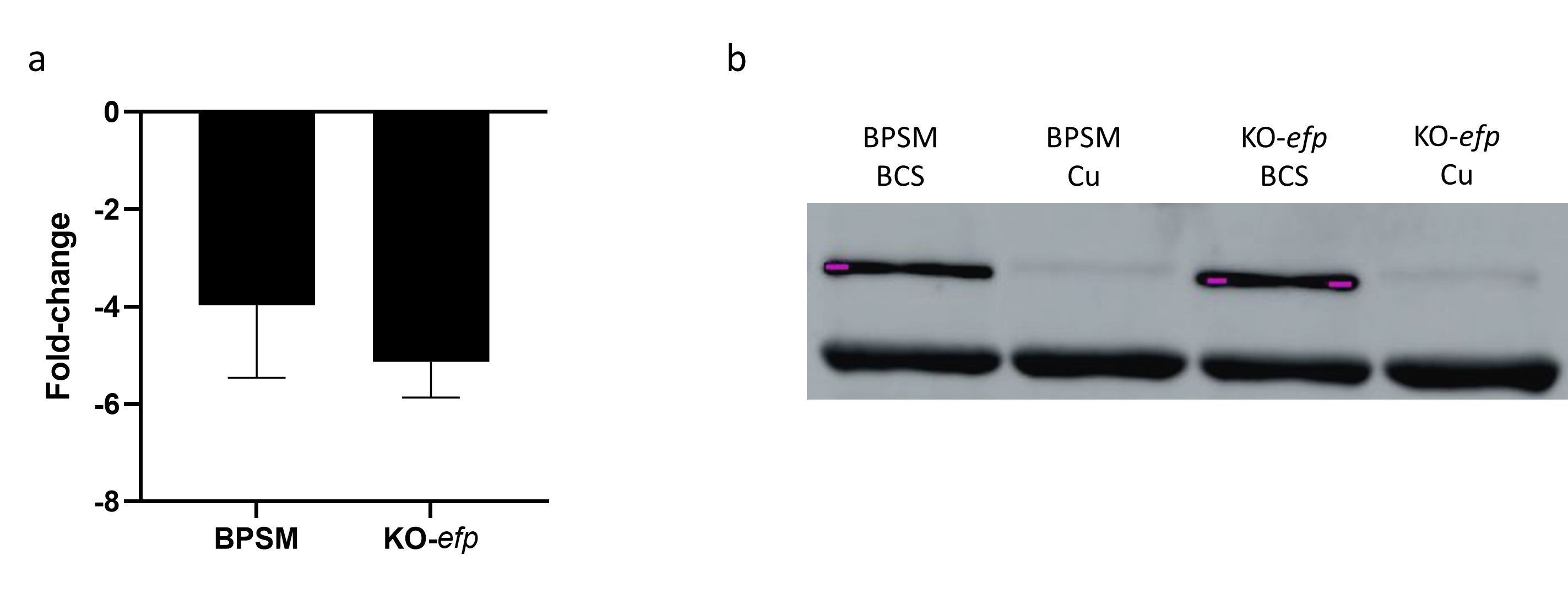

Supplement: FIG S4 [file mbio.00912-22-s0004.tif]

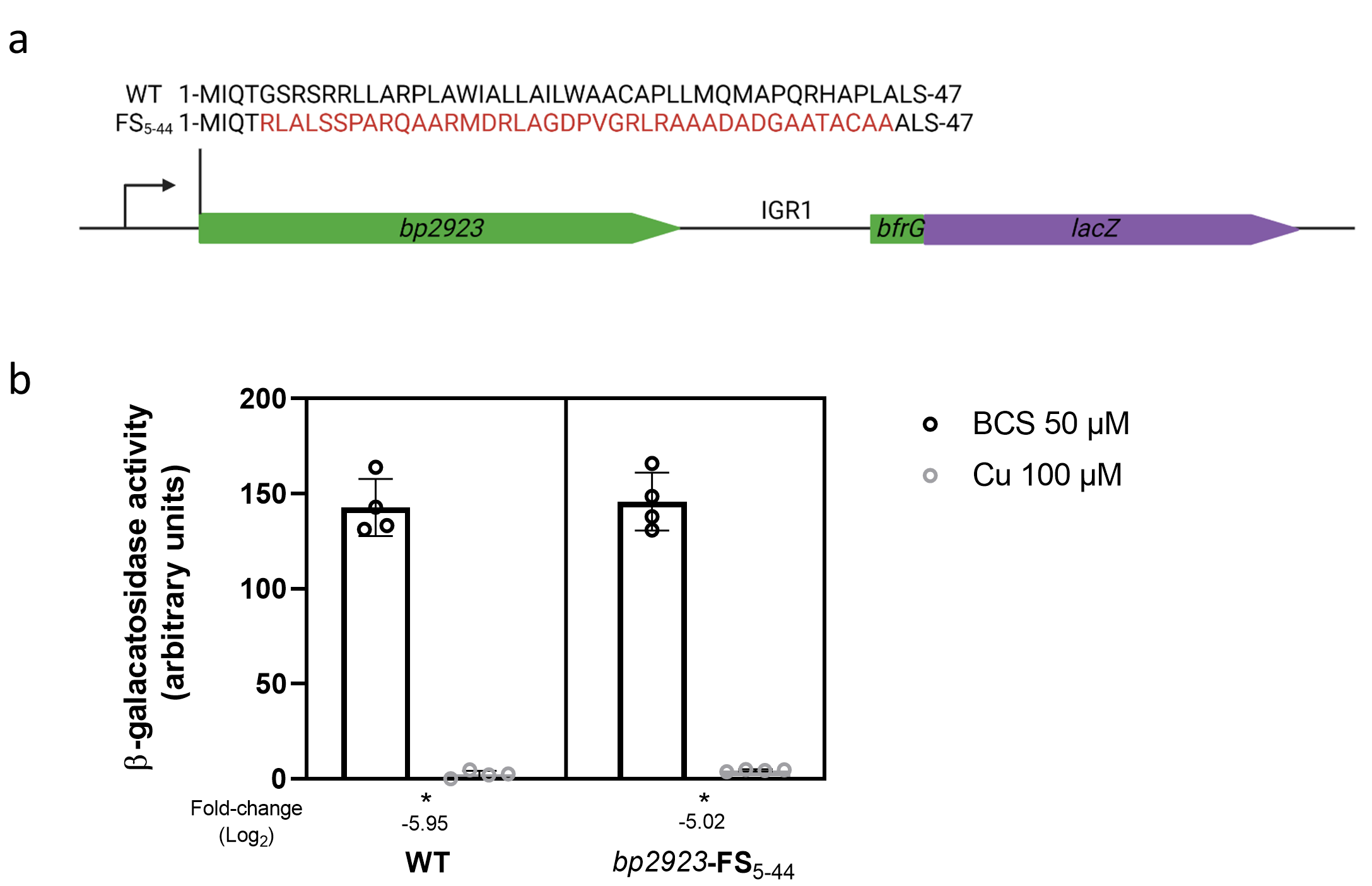

Supplement: FIG S5 [file mbio.00912-22-s0005.tif]
