# Supplementary material for: Posttranscriptional Regulation by Copper with a New Upstream Open Reading Frame
Source: mBio. 2022 Jul 13;13(4):e00912-22. doi: 10.1128/mbio.00912-22 (PMC9426467; doi:10.1128/mbio.00912-22)
Supplement: TABLE S2 [file mbio.00912-22-s0007.docx]

**Table S2.** **Plasmids used in this study**

|  | Plasmid name | Description |
| --- | --- | --- |
|  | pSS1129 | Suicide plasmid for homologous recombination in *Bordetella* |
|  | pQC2123 | Suicide plasmid carrying *lacZ* for chromosomal transcriptional fusions in *B. pertussis* |
|  | pRM1 | Suicide plasmid for genetic insertion in the *ure* locus of *Bordetella pertussis* |
| Allelic exchange | pSS1129-∆*bp2923* | Plasmid carrying the regions flanking *bp2923* for its deletion |
|  | pSS1129-∆*bfrG* | Plasmid carrying the regions flanking *bfrG* for its deletion |
|  | pSS1129-*bp2923*-OCU | Plasmid carrying *bp2923-*OCU and its flanking regions for insertion in BP*Δ2923* by homologous recombination |
|  | pRM1-*bp2923* | Plasmid carrying *bp2923* flanked by *ure* locus regions for complementation in *trans* |
| Gene KO | pFUS2-efp | Suicide plasmid carrying a 316-bp fragment of *efp* used to knock out this gene |
| Chromosomal translational *lacZ* fusions | pQC2123-*bp2923-lacZ* | Translational fusion between the first 10 codons of *bp2923* and *lacZ*; contains a 631-bp EcoRI-XhoI fragment in pQC2123 for homologous recombination |
|  | pQC2123-*bfrG-lacZ* | Translational fusion between the first 10 codons of *bfrG* and *lacZ*; contains a 1225-bp EcoRI-XhoI fragment in pQC2123 for homologous recombination |
|  | pQC2123-*bp2921-lacZ* | Translational fusion between the first 10 codons of bp2921 and *lacZ*; contains a 635-bp EcoRI-XhoI fragment in pQC2123, for homologous recombination |
|  | pUC57-*cruR* wt | Plasmid carrying a 797-bp EcoRI-XhoI fragment encompassing part of *bp2924,* the intergenic region between it and *cruR,* *cruR* and the 10 first codons of *bfrG,* used to introduce mutations in *bp2923* |
|  | pQC2123-*bp2923*P_120_A+P_121_A*-lacZ* | Translational fusions between the first 10 codons of *bfrG and lacZ,* with the indicated mutations in *bp2923; the* EcoRI-XhoI fragments are from the corresponding pUC57-*cruR* plasmids. The fragment comprising *bp2924* and the intergenic region between it and *bp2923* drives homologous recombination in the chromosome of BP*Δ2923* |
|  | pQC2123-*bp2923*P_141_A+P_142_A-*lacZ* |  |
|  | pQC2123-*bp2923*C_90_S+C_93_S-*lacZ* |  |
|  | pQC2123-*bp2923*Y_50_STOP-*lacZ* |  |
|  | pQC2123-*bp2923*R_139_A+A_140_S-*lacZ* |  |
|  | pQC2123-*bp2923*C_51_S-*lacZ* |  |
|  | pQC2123-*bp2923*FS_117-123_-*lacZ* |  |
|  | pQC2123-*bp2923*FS_5-44_-*lacZ* |  |
|  | pQC2123-*bp2923*W_133_STOP-*lacZ* |  |
|  | pQC2123-*bp2923*-2-*lacZ* |  |
|  | pQC2123-*bp2923*-1-*lacZ* |  |
|  | pQC2123-*bp2923*+1-*lacZ* |  |
|  | pQC2123-*bp2923*+2-*lacZ* |  |
|  | pQC2123-*bp2923*+5-*lacZ* |  |
|  | pQC2123-*bp2923*-OCU-*lacZ* |  |
