# Supplementary material for: Posttranscriptional Regulation by Copper with a New Upstream Open Reading Frame
Source: mBio. 2022 Jul 13;13(4):e00912-22. doi: 10.1128/mbio.00912-22 (PMC9426467; doi:10.1128/mbio.00912-22)
Supplement: TABLE S3 [file mbio.00912-22-s0008.docx]

**Table S3.** **Oligonucleotides used in this study**

|  |  | Sequences (5'-3') |
| --- | --- | --- |
| Deletions | 2923 EcoRI UP | ATGAATTCACGCCGTAGCATTTCTCCATGT |
|  | 2923 XbaI LO | ATTCTAGAGCCGGTCTGGATCATGGT |
|  | 2923 XbaI UP | TATCTAGATGAGCGCGGCAGCCTTCC |
|  | 2923 HindIII LO | ATAAGCTTCTCGCGGTTGATGACGGTCAC |
|  | bfrg UP | GGATCCCAGGAGCAATCGCTTCCCGT |
|  | bfrG XbaI LO | TGTAGCGGTAGGTCTCGAGGT |
|  | bfrG XbaI UP | ACCTCGAGACCTACCGCTACA |
|  | bfrG LO | TGAAGCTTCGCCTGCTCCCTCAGAAGC |
| *bp2923* complementation | 2923comp UP | TAGGATCCCGCGTCAGTCACGGGCAAGG |
|  | 2923comp LO | TATCTAGAAAGGCTGCCGCGCTCAGG |
| Gene knock out | efp UP | ATAAGCTTGACCCGCTCGTCGTCCAGA |
|  | Efp LO | TAGGATCCGCCGTCGTAGAAGACCACCT |
| 5'RACE | bfrG | GCCAGCGCCAGTCCGACC |
|  | 2923-OCU | CTCCGTGCAGTAGTCGACCGAT |
|  | 2923-OCU nested | ATGGTCCGAGATTATAAACAGCG |
| *lacZ* translational fusions | Fus-UP | ATGAATTCTCAAGACCCGGTC |
|  | 2923Fus LO | ATCTCGAGCAGGCGACGAGAGCGCGA |
|  | bfrGFus LO | ATCTCGAGGGCGTAGTAAAGGGAAGTCTGT |
|  | 2921Fus UP | TAGAATTCACCACCGGCACCCAGGTCGAG |
|  | 2921Fus LO | TACTCGAGGGCGCGCAAGACTAGGGTCGT |
| RT-PCR | RT UP | ACCTGGAACATTGCCCGTTCT |
|  | RT LO | CACGACCACCGGCGCGAGTTG |
| qRT-PCR | *bp3416* UP | GTTCCTGGAAGTGCTGCTG |
|  | *bp3416* LO | GATGTCGAAGGCATTCTGG |
|  | *bp2923* UP | CCGCTCGCATGGATCGCCTT |
|  | *bp2923* LO | CGTCCCCTGCTCGGTGCAAT |
|  | *bfrG* UP | CTCGACACGCAGGAAATCGC |
|  | *bfrG* LO | CAACTGGCTGACCTGCGAAC |
|  | *bp2921* UP | CAAGCGCTGGCTGTATTTG |
|  | *bp2921* LO | CAGATTGGGATAGCCGACATAC |
| Mutagenesis | G5+1 UP | GGACCATGATCCAGACCAGGCTCGCGCTCTCGTCG |
|  | G5+1 LO | CGACGAGAGCGCGAGCCTGGTCTGGATCATGGTCC |
|  | L44-1 UP | CAACGGCATGCGCCGCTGCGCTGTCGGTCGACTA |
|  | L44-1 LO | TAGTCGACCGACAGCGCAGCGGCGCATGCCGTTG |
|  | C51S UP | CGCTGTCGGTCGACTATAGCACCGAGCAGGGGACG |
|  | C51S LO | CGTCCCCTGCTCGGTGCTATAGTCGACCGACAGCG |
|  | 116+1 UP | CTTGCCGGCGCGCGCCGATTTCCTATCC |
|  | 116+1 LO | GGATAGGAAATCGGCGCGCGCCGGCAAG |
|  | 124-1 UP | CGATTTCCTATCCTCCTCTCTTTACGTTGCCCG |
|  | 124-1 LO | CGGGCAACGTAAAGAGAGGAGGATAGGAAATCG |
|  | W133* UP | CGTCGCAGCATGCCTGAACCGGCGCGCAGCCGC |
|  | W133* LO | GCGGCTGCGCGCCGGTTCAGGCATGCTGCGACG |
| In vitro transcription | FWT1b | TTATCAAAAAGAGTATTGACTTAAAGTCTAACCTATAGGATACTTACAGCCAGATTGTGCGCGAGCGTGGG |
|  | FWT2b | TTATCAAAAAGAGTATTGACTTAAAGTCTAACCTATAGGATACTTACAGCCAGGGCGACCTGGCCC |
|  | FWT3b | TTATCAAAAAGAGTATTGACTTAAAGTCTAACCTATAGGATACTTACAGCCAGCATGCCTGGACCGGCGCGC |
|  | FWT4-6 | TTATCAAAAAGAGTATTGACTTAAAGTCTAACCTATAGGATACTTACAGCCAGGGCGACCTGGCCC |
|  | REVT1-3 | CGAGGGCGTAGTAAAGGGAAGTCTG |
|  | REVT4 | CGGCGGGCATGTCGATGACGGGC |
|  | REVT5 | GGCCCGGCCGCAAGGCGGACGGGC |
|  | REVT6 | GCACGGCTGGCCGTGGGCGCTGG |
